# Affective Fear of Crime and Its Association with Depressive Feelings and Life Satisfaction in Advanced Age: Cognitive Emotion Regulation as a Moderator?

**DOI:** 10.3390/ijerph18094727

**Published:** 2021-04-29

**Authors:** Nadezhda Golovchanova, Katja Boersma, Henrik Andershed, Karin Hellfeldt

**Affiliations:** School of Law, Psychology and Social Work, Örebro University, SE-701 82 Örebro, Sweden; katja.boersma@oru.se (K.B.); henrik.andershed@oru.se (H.A.); karin.hellfeldt@oru.se (K.H.)

**Keywords:** fear of crime, mental health, depressive feelings, emotion regulation, well-being, life satisfaction

## Abstract

Fear of crime is a substantial problem for older adults and is associated with reduced subjective well-being. However, less is known about factors that could moderate the associations between fear of crime and mental health problems and well-being in advanced age. Cognitive emotion regulation could serve as a potentially buffering factor for adverse health outcomes related to fear of crime due to its potential importance in managing feelings when facing threatening situations. The current study investigated the associations between affective fear of crime with depressive feelings and life satisfaction and examined whether adaptive and maladaptive cognitive emotion regulation strategies moderated these associations in a sample of older adults (age 64–106) in Sweden (N = 622). The results showed that affective fear of crime was associated with more depressive feelings, less life satisfaction, and more frequent use of such maladaptive cognitive emotion regulation strategies as rumination, catastrophizing, and blaming others. Moreover, rumination and self-blame moderated the associations between affective fear of crime and life satisfaction. Adaptive emotion regulation strategies were not associated with affective fear of crime and did not decrease the strength of its association with depressive feelings and with life satisfaction. These findings allow us to conclude that maladaptive emotion regulation could be considered a vulnerability factor in the association of fear of crime with life satisfaction.

## 1. Introduction

Perceived safety is an important element of well-being while aging. Fear of crime, indicating a lack of subjective safety, has been associated with reduced well-being for older adults [1,2,3] as well as reduced mobility [4,5,6]. While maintaining high quality of life of older adults is an important ultimate goal within the recent understanding of successful aging [7], increasing our knowledge on factors that hinder quality of life elements in advanced age is crucial. Because feeling safe and secure is among the central elements of quality of life while aging [8,9], the association between fear of crime as a factor of unsafety and aspects of well-being in advanced age warrants more research attention.

Although fear of crime can potentially undermine quality of life in older adults, little is known about factors that could decrease or strengthen the associations between fear of crime, mental health problems, and subjective well-being in advanced age. Here, cognitive emotion regulation deserves special attention because of its potential importance in managing feelings when facing threatening situations [10]. Cognitive emotion regulation refers to the conscious cognitive appraisal of a stressful situation to manage an emotional arousal [10,11]. In advanced age, cognitive emotion regulation has been shown to be an important mechanism related to maintaining higher levels of well-being when facing difficulties in various life domains [12,13]. Recent research showed that accommodative coping (i.e., an emotion regulation strategy that includes flexible adjustment to difficult life circumstances, as well as re-evaluation and positive reappraisal of a threatening situation) reduced the strength of the association between fear of crime and depressive symptoms in a sample of adults [14]. However, the role of various dimensions of cognitive emotional regulation in experiencing fear of crime and its links with subjective well-being indicators in older adults is yet to be investigated. Therefore, the current study addressed this important knowledge gap by investigating the role of various adaptive and maladaptive cognitive emotional regulation strategies as potential moderators in the associations between fear of crime with depressive feelings and with life satisfaction, respectively. 

### 1.1. Fear of Crime in Advanced Age

Fear of crime is a multifaceted phenomenon that is often described as consisting of affective, cognitive, and behavioral dimensions [14,15,16,17]. In the current study, we rely on the definition of fear of crime as “the negative emotional reaction generated by crime or symbols associated with crime”, proposed by LaGrange and Ferraro [18] p. 73. Thus, in line with previous research that operationalized fear of crime as worry about crime (e.g., [19,20,21]), we focus on this affective dimension of fear of crime, i.e., worry about crime and crime threats as the most closely associated with emotion regulation processes. 

Another important consideration for the assessment of fear of crime is considering the fear of different types of crime [18,22,23,24]. A national survey in Sweden demonstrated, for example, that older adults were particularly fearful of being burgled, robbed, or attacked [25]. Nevertheless, there may be safety concerns that are specifically relevant for older people [26]. For instance, older adults could perceive others who have access to their home as a threat, and this might result in being fearful of an offense potentially committed by these individuals. For those who receive assistance with daily tasks at home, the very presence of another person could potentially be provoking such fear. Furthermore, another distinct fear is altruistic fear of crime, which refers to fear that a close person would be victimized [27]. Such altruistic fear might be particularly relevant for older adults given their general high awareness of victimization of others, which can elevate concerns related to others’ safety [26]. Considering that multiple-item indicators are considered beneficial for fear of crime assessment [28], we argue for the need to include these aspects into the measurement when examining the affective (emotional) fear of crime in older adults. 

### 1.2. Fear of Crime, Depressive Feelings, and Life Satisfaction

The conceptual literature on fear of crime emphasizes the negative mental health outcomes associated with experiencing fear of crime [29,30,31,32]. In advanced age, experiencing fear of crime has been linked with anxiety, depression, and negative affect [1]; anxiety, stress, and suicide attempts [33]; and anxiety and depression [2]; and depressive symptoms [1,3]. Bi-directional associations between fear of crime and mental health in advanced age have been proposed [31]. In the current study, we investigate the association between fear of crime and well-being indicators while considering the hypothesized reduced well-being to, theoretically, be an effect of fear of crime. In line with previous studies that examined the association of fear of crime with mental health aspects and life satisfaction [3,34,35], we further explore these associations between fear of crime and well-being indicators in advanced age.

In the range of mental health problems associated with fear of crime in advanced age, depression is especially important because of its high prevalence among older adults [36,37]. Although some studies approached depressive feelings as an inverse indicator of psychological well-being (e.g., [14]), current approaches to well-being in advanced age stress the importance of differentiating between well-being and problematic mental health outcomes. Mental health aspects and well-being have been shown to belong to different dimensions of health indicators in an overall evaluation of positive health [38], which might suggest certain differences in factors maintaining or undermining these health dimensions. Moreover, the absence of negative well-being outcomes does not automatically imply higher levels of positive indicators of well-being [8]. More specifically, absence of depressive feelings in advanced age does not directly equate to high levels of life satisfaction. An increasing amount of empirical research provides evidence of negative associations between fear of crime and life satisfaction [34,35]. Therefore, in the current study we address both depressive feelings and life satisfaction as relevant for fear of crime and explore the associations of fear of crime with depressive feelings and with life satisfaction separately. 

Although the general link between fear of crime with depressive feelings and with life satisfaction has been well established, still very little is known concerning factors that could mitigate these relations. As an example, one study was able to identify a subgroup of individuals reporting taking behavioral precautions as a protective mechanism, which reduced the effect of fear of crime on life quality to minimal or none [19]. Inspired by this reasoning, in the current study, we investigated a potential role of another protective mechanism. We examined whether adaptive cognitive emotion regulation would decrease the strength of the association of fear of crime with depressive feelings and life satisfaction. Additionally, we explored whether maladaptive cognitive emotion regulation increased the strength of the association of fear of crime with depressive feelings and life satisfaction.

### 1.3. Fear of Crime, Vulnerability, and Cognitive Emotion Regulation

According to the vulnerability perspective, differences in experiences of fear of crime can be explained by vulnerability factors, resulting in some individuals or groups of individuals being more susceptible to worries about crime [39,40]. In line with this theorical perspective, vulnerability should be understood as a feeling of having little or no control over the likelihood or the consequences of a potential crime. Research on fear of crime within the vulnerability perspective has devoted significant attention to physical and social vulnerability factors, such as female gender, higher age, health limitations, socioeconomic status, or level of education [20,39,40,41,42,43]. Considerably less research has addressed psychological vulnerability factors for experiencing fear of crime [44] although vulnerability refers not only to objective factors but also to the feeling of loss of control [16]. However, existing research has shown that individual psychological vulnerability factors seem important in explaining the experience of fearing crime. For instance, trait anxiety [45], phobic disorders [46], personality traits [47], and loneliness [48] have been associated with fear of crime, and fear of crime itself has been considered to be a personal disposition [49]. Following the logic of the vulnerability perspective, these studies show the importance of examining other psychological factors that could also potentially serve as hypothetical vulnerability factors, explaining how fear of crime relates to ill-health.

An additional factor of psychological vulnerability in relation to fear of crime is cognitive emotion regulation [14]. When fear of crime is approached in its affective facet, i.e., as a feeling or worry about crime, we can presume that to a certain extent, this affective reaction can be down- or upregulated by an individual. Various cognitive emotion regulation strategies for managing undesired emotional states have been identified. Examples of such cognitive emotion strategies are positive refocusing, blaming others, catastrophizing, acceptance, etc. These strategies can be further conceptualized as being more or less adaptive as indicated by their associations with mental health problems [10,11].

While cognitive emotion regulation is generally recognized as a factor contributing to well-being, its role in the well-being of older adults is especially important. In advanced age, cognitive emotion regulation is assumed to be an important mechanism related to maintaining higher levels of well-being despite frequent and often unavoidable losses in various life domains [12,13]. However, both strengths and vulnerabilities in age-related emotion regulation are important to consider when explaining differences in the well-being of older adults [50]. Therefore, we expect that adaptive cognitive emotion regulation might buffer the strength of fear of crime or its associations with aspects of well-being, whereas maladaptive emotion regulation strategies would be an additional individual vulnerability factor in managing fear of crime. In the current study, we tested whether being prone to blaming oneself, ruminating, blaming others, and catastrophizing is positively related to affective fear of crime. We also examined whether these maladaptive cognitive emotion regulation strategies strengthen the hypothesized positive association of fear of crime with depressive feelings and increase the strength of the hypothesized negative association between affective fear of crime and life satisfaction. On the other hand, we explored whether using adaptive cognitive emotion regulation strategies such as acceptance, positive refocusing, and putting into perspective helps to downregulate the emotional response to a perceived criminal threat and whether these strategies altered the strength of the association between fear of crime and depressive feelings and life satisfaction.

### 1.4. Current Study

As summarized above, very few studies have investigated the role of cognitive emotion regulation in the associations between fear of crime with depressive feelings and life satisfaction. Even less is known about the role of adaptive and maladaptive cognitive emotion regulation strategies in these associations. The current study aimed to address this research gap by investigating the association between affective fear of crime and depressive feelings and life satisfaction among older adults and exploring whether adaptive (acceptance, positive refocusing, and putting into perspective) and maladaptive (self-blame, rumination, blaming others, and catastrophizing) cognitive emotion regulation strategies moderated these associations in older adults. The following research questions were formulated:

1. What are the associations between affective fear of crime and depressive feelings, on the one hand, and life satisfaction, on the other hand, for older adults, while controlling for age and gender?

**Hypothesis** **1** **(H1).** *We expected a positive association between fear of crime and depressive feelings, and a negative association between fear of crime and life satisfaction.*

2. What is the association between affective fear of crime and adaptive and maladaptive cognitive emotion regulation strategies in advanced age, while controlling for age and gender?

**Hypothesis** **2** **(H2).** *We expected a negative association of adaptive emotion regulation strategies with affective fear of crime and a positive association of maladaptive emotion regulation strategies with affective fear of crime.*

3. Do adaptive and maladaptive cognitive emotion regulation strategies moderate the relation between fear of crime and depressive feelings, and fear of crime and satisfaction with life, while controlling for age and gender?

**Hypothesis** **3** **(H3).** *Less-adaptive cognitive emotion regulation strategies were hypothesized to be a vulnerability factor for experiencing fear of crime and, thus, were expected to strengthen the association of affective fear of crime with depressive feelings and life satisfaction. More-adaptive emotion regulation strategies were hypothesized to play a protective role and, thus, to decrease the strength of the association of affective fear of crime with depressive feelings and life satisfaction.*

## 2. Materials and Methods

### 2.1. Participants and Procedure 

The current study employed the 65+ and Safe Study data, which was collected in a mid-sized Swedish municipality in 2019. This is a cross-sectional survey project focused on safety and the experience of fearing crime among older adults living in senior apartments. Thus, older adults who (1) were 65 years old in 2019 or older, (2) were residents of a senior apartment belonging to Örebro bostäder AB (ÖBO)—a Swedish municipal housing company, and (3) did not have severe cognitive impairment were invited to participate in the study. These senior apartments are in essence regular rental apartments, not assisted living or nursing home accommodations. The apartments are available for residents who are 65 years and older and, thus, are equipped with specific design elements (e.g., elevators, automatic door openers, etc.). The apartments are located in various neighborhoods of the municipality, including urban and rural areas, and differ in their size. The project gained approval from the Swedish Ethical Review Agency (Dnr: 2019-02248).

The study sample included 622 participants (response rate 49.5%). Responders did not significantly differ from the non-responders with regard to gender, χ^2^ (2, N = 1237) = 1.17, *p* = 0.56. However, the non-responders were older compared to those who responded, *t* (1220) = −3.41, *p* = 0.001. The average age of the respondents was 77.6 (age range 64–106), and 60.6 % were female. In this sample, 68.5% reported having education equivalent to a high school degree or lower, 36.7% were married, 7.7% lived together with a partner, 2.4% lived separately with a partner, 15.8% were divorced, 9.6% were single, and 26.4% were widowed. Further, 23.5% received assistance with daily tasks at home (e.g., with grocery shopping, household tasks, etc.), and 4.9% reported being a victim of a crime during the last year.

### 2.2. Measures

#### 2.2.1. Independent Variable

Fear of crime (affective component) was assessed by six single items each inquiring whether the fear of a specific crime was experienced by the respondents during the last year (e.g., “Has it happened in the past year that you have been worried that you will be robbed?”). The specific crimes included the fear of break-in, the fear of being attacked or assaulted, the fear of being robbed, the fear of rape or a sexual assault, fear of crime committed by those who have access to the apartment, and the altruistic fear of crime. The response options for each item ranged from “Never” (1) to “Very often” (5). The affective fear of crime index was calculated using a mean score of the six items (Cronbach’s alpha = 0.80).

#### 2.2.2. Dependent Variables

Life satisfaction was assessed with the Satisfaction with Life Scale (SWLS) [51]. The scale consists of five items each rated on a 7-point Likert scale, from “Strongly disagree” to “Strongly agree” (e.g., “The conditions of my life are excellent”, “I am satisfied with my life”). The life satisfaction scale was created using the mean score of all the five items (Cronbach’s alpha = 0.88). 

Depressive feelings were assessed with the depression subscale (HADS-D) of the Hospital Anxiety and Depression Scale (HADS) [52]. The subscale consists of seven items each rated on a 4-point Likert scale from 0 to 3 (e.g., “I feel as if I am slowed down”, “I feel cheerful”). The depressive feelings scale was created using the mean score of at least six of the total seven items (Cronbach’s alpha = 0.74).

#### 2.2.3. Potentially Moderating Variables

Cognitive emotion regulation strategies were assessed with the respective subscales of the short version of the Cognitive Emotion Regulation Questionnaire (CERQ-short) [11]. In line with the questionnaire’s instruction [10], the participants were asked to evaluate their general thoughts when being exposed to negative or unpleasant events. The nine subscales that CERQ contains are self-blame, acceptance, rumination, positive refocusing, refocus on planning, positive reappraisal, putting into perspective, catastrophizing, and blaming others. Each subscale is represented by two items rated on a 5-point Likert scale from “Almost never” (1) to “Almost always” (5). Examples of items are “I continually think how horrible the situation has been” for catastrophizing; “I am preoccupied with what I think and feel about what I have experienced” for rumination; and “I think of something nice instead of what has happened” for positive refocusing. 

Confirmatory factor analysis revealed that the refocus on planning and positive reappraisal subscales did not confirm their expected factor structure in our sample; therefore, these two subscales were excluded from the analyses. Cronbach’s alphas for the seven subscales included in the analyses were 0.69 (catastrophizing), 0.70 (blaming others), 0.59 (rumination), 0.59 (self-blame), 0.76 (acceptance), 0.67 (positive refocusing), and 0.65 (putting into perspective). Each cognitive emotional regulation strategy scale was created using the mean score of the two items included in the subscale. Based on the previous conceptualization and associations with mental health outcomes, acceptance, positive refocusing, and putting into perspective are regarded as more-adaptive cognitive emotion regulation strategies, whereas catastrophizing, blaming others, rumination, and self-blame are regarded as less-adaptive cognitive emotion regulation strategies [10]. 

### 2.3. Covariates

#### 2.3.1. Age

Mixed evidence exists on whether fear of crime increases with age [53]. Interestingly, studies assessing different aspects of fear of crime separately showed that it tends to increase with age only in its behavioral aspect [15,16]. Although we address the affective component of fear of crime in the current study, we find it important to control for age as the sample contains a wide age range (64–106) and significantly less is known about affective fear of crime experience and its links with well-being outcomes in the oldest ages. Age was assessed as a continuous variable.

#### 2.3.2. Gender

A body of research suggests that women are more fearful of crime than men are [54,55]. Moreover, gender plays a role in the associations between fear of crime and well-being indicating stronger associations with certain negative well-being outcomes for men than for women [33,48]. Considering these previous findings, we controlled for gender in the current study (the gender variable was coded as 1 = female, 2 = male). 

### 2.4. Analytic Strategy

Initially and to answer the first two research questions, Pearson zero-order correlation coefficients and partial correlations (controlling for age and gender) were calculated to test the associations between the main variables, i.e., affective fear of crime, depressive feelings, life satisfaction, adaptive (acceptance, positive refocusing, and putting into perspective) and maladaptive (catastrophizing, blaming others, rumination, and self-blame) cognitive emotion regulation strategies. 

For the third research question, the moderation effect was tested by means of multiple regression analysis. Separate multiple regression models were tested in which depressive feelings and life satisfaction served as outcomes, and for each of the seven emotion regulation strategies separately, while controlling for age and gender. In total, fourteen regression models were run (seven with depressive feelings serving as outcome, seven with life satisfaction serving as outcome). As such, in each of the regression models using depression or life satisfaction as outcome, age, gender, affective fear of crime, one of the seven cognitive emotional strategies as well as the related interaction term was entered as independent variables. As recommended, continuous variables (i.e., affective fear of crime and each of the seven emotion regulation strategies) were centered prior to being entered in analyses [56]. In total, seven interaction variables were created using the centered variables: affective fear of crime index × emotional regulation strategy. Statistical significance of the interaction term indicated the presence of the moderation effect. Multicollinearity was assessed by inspecting the tolerances considering values approaching zero as indicative of multicollinearity [57]. No multicollinearity problems were detected in any of the models. (Since one outcome variable (depressive feelings) was not normally distributed (skewness = 1.028; kurtosis = 1.280), as an alternative, we also ran the same regression models with this outcome standardized, as well as a series of logistic regression models with results showing no noteworthy differences (results available upon request). The life satisfaction variable did not display severe deviation from the normality assumption (skewness = −0.563; kurtosis = −0.221).) All analyses were carried out in IBM SPSS 27 (IBM, Armonk, NY, USA). Significant interaction effects were plotted by means of PROCESS v3.5. Conditional values of the moderator were set at the 16th, 50th, and 84th percentiles. These values represent the distribution of the moderator variable (cognitive emotion regulation strategies) and indicate low, moderate, and high levels of the moderator. Such choice of conditional values is preferred when moderator variables are not perfectly normally distributed [58].

## 3. Results

### 3.1. Descriptive Statistics

Descriptive statistics of the study variables are presented in Table 1. The mean level of affective fear of crime in our sample is comparable to that reported for adults (e.g., [23]). Concerning depression, according to the recommended cutoff scores [59], 12.5% of participants demonstrated mild, moderate, or severe depression, which is within the estimation that 8% to 16 % of older adults living in a community present depressive symptoms [36]. The life satisfaction mean score is within the range of previously reported values in studies on older adults (e.g., [60]).

### 3.2. Fear of Crime, Depressive Feelings, Life Satisfaction, and Cognitive Emotion Regulation

Zero-order and partial correlations (controlling for age and gender) among the study variables were performed to test hypotheses one and two (Table 2). In line with the first hypothesis, the affective fear of crime index was positively correlated with depressive feelings and was negatively correlated with life satisfaction. Further, affective fear of crime was positively correlated with rumination, blaming others, and catastrophizing, thus demonstrating that those who use these strategies more frequently experience more fear of crime. However, there were no associations between affective fear of crime and self-blame, acceptance, positive refocusing, or putting into perspective. These results are in line with the second hypothesis for most of maladaptive emotion regulation strategies and are contrary to the hypothesis for adaptive emotion regulation strategies.

### 3.3. Moderating Role of Cognitive Emotion Regulation Strategies

A series of hierarchical regression analyses with depressive feelings and life satisfaction as dependent variables was performed. For each of the two dependent variables, seven models were tested separately for the significance of the interaction effect of each of the cognitive emotion regulation strategies with overall affective fear of crime, while controlling for age and gender.

#### 3.3.1. Depressive Feelings

Results of the hierarchical regression analyses with affective fear of crime, various cognitive emotional regulation strategies, and related interaction term as independent variables, and with depressive feelings as dependent variable are presented in Table 3. Contrary to the first part of hypothesis three, none of the interaction terms had statistically significant effects on the outcome, thus showing no moderation effect of any of the seven cognitive emotion regulation strategies on the relationship between fear of crime and depressive feelings.

#### 3.3.2. Life Satisfaction

**Figure 1:**
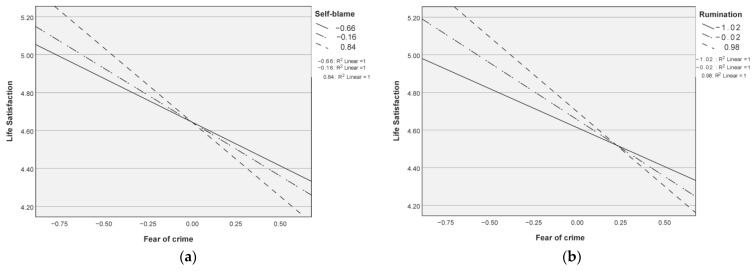
Moderating effect of cognitive emotion regulation strategies on the association of affective fear of crime and life satisfaction: (**a**) self-blame; (**b**) rumination.

Similar to the hierarchical regression models predicting depressive feelings, a series of models with affective fear of crime, cognitive emotional regulation strategies, and related interaction term as independent variable and with life satisfaction as dependent variable were tested. Results are presented in Table 4 and indicate a significant effect of the interaction in the models with self-blame and rumination, thus demonstrating the moderation effect of these cognitive emotion regulation strategies on the association between fear of crime and life satisfaction. As can be seen in Figure 1, participants demonstrating higher levels of self-blame and rumination, combined with higher levels of fear of crime, display significantly lower levels of life satisfaction. The conditional effects at all levels of moderators were statistically significant (*p* < 0.01) both for self-blame and rumination, indicating significant differences in the slopes at different levels of these moderators. Thus, the second part of hypothesis three was confirmed for self-blame and rumination in advanced age, since the two increased the strength of the negative association between affective fear of crime and life satisfaction. However, no moderation effect of cognitive emotion regulation was observed in the models with blaming others, catastrophizing, acceptance, positive refocus, and putting into perspective.

## 4. Discussion

The current study investigated the associations between affective fear of crime and well-being outcomes in advanced age, as well as the potential role of cognitive emotion regulation strategies as moderators of these associations. As hypothesized regarding the first research question, affective fear of crime was positively associated with depressive feelings and negatively associated with life satisfaction. These associations remained significant when controlling for age and gender. This is in line with previous research on the associations of fear of crime with negative mental health and well-being outcomes obtained in samples of adults [34,35,61] and older adults [1,3]. These results highlight the importance of fear of crime as a factor associated with reduced subjective well-being in advanced age. 

The second research question explored the associations between affective fear of crime and seven cognitive emotion regulation strategies in advanced age, namely, self-blame, rumination, blaming others, catastrophizing, acceptance, positive refocus, and putting into perspective. In line with our hypothesis, those older adults who used rumination, blaming others, and catastrophizing (maladaptive cognitive emotion regulation strategies) also experienced more fear of crime, and these associations were significant when controlling for age and gender. However, there were no associations between affective fear of crime and self-blame. Relying on rumination and catastrophizing may be linked to more affective fear of crime by means of increased thought focus on threats of crime and their negative consequences. More frequent use of blaming others in case of stressful events might be linked to heightened fear of crime by focusing on the external nature of the perceived criminal threat: it is the “other”, the potential offender that is a perceived cause of the threat. Consequently, such negative thought processes may result in feeling helpless and vulnerable in the face of potential threats, thus enhancing the potential victims of crime’s self-perception and hindering feelings of agency and competence in withstanding crime [62]. Therefore, such cognitive emotion regulation strategies as rumination, catastrophizing, and blaming others can be considered to be individual psychological vulnerability factors for experiencing fear of crime. 

Contrary to our expectations, there were no associations between affective fear of crime and acceptance, positive refocus, and putting into perspective. Thus, none of the adaptive emotion regulation strategies were associated with affective fear of crime. Therefore, based on the current study, adaptive cognitive emotion regulation strategies cannot be considered as a protective factor for the experience of fear of crime. One explanation for this might be that fear of crime, unlike some other self-related fears in advanced age (e.g., fear of falling or fear of dementia), includes a strong environmental component in a sense that criminal threats are external to an aging person. Regulating emotions only on the cognitive level may not be sufficient for managing fear of crime, and certain behavioral regulation strategies may play a more decisive role in this (e.g., avoiding situations when one might feel fearful, moving to a safer neighborhood). Previous research has shown that certain individuals, when experiencing fear of crime, take behavioral precautions and report that fear of crime does not impact their quality of life [19]. Because older adults were previously shown to engage in protective and avoiding behavior as a response to fear of crime compared to younger adults [15,16], further research should explore the role of such behavior in potentially reducing the effect of fear of crime on well-being. 

The third research question tested the moderation effect of cognitive emotion regulation strategies in the associations between fear of crime and well-being outcomes. We observed a moderation effect of rumination and self-blame on the link between overall affective fear of crime and life satisfaction, indicating that these cognitive emotion regulation strategies might be individual psychological vulnerability factors strengthening the association of fear of crime and life satisfaction. In other words, being prone to focus on thoughts about a stressful situation and holding others responsible for unfortunate events combined with affective fear of crime was associated with significantly lower levels of life satisfaction. A somewhat unexpected finding is that we observed no moderation effect of maladaptive cognitive emotion regulation strategies in the link between overall fear of crime and depressive feelings. However, the differences in patterns of moderation effects between the models with depressive feelings and life satisfaction as the outcome highlight the differences between these well-being indicators and the factors important for their maintenance. 

Contrary to our hypothesis, there was no moderation effect of adaptive cognitive emotion regulation strategies either in the association between overall affective fear of crime and depressive feelings or in that between fear of crime and life satisfaction. Thus, these results demonstrated that acceptance, positive refocus, and putting into perspective do not decrease the strength of the association of affective fear of crime either with depressive feelings or with life satisfaction. This result differs from previous findings showing a protective effect of accommodative coping in the association between fear of crime and depressive feelings in a sample of adults high on victimization experience [14]. A possible explanation for this discrepancy could be the sample differences, with our sample being low on victimization experience and significantly higher in age. Another plausible explanation can also lie in the specific fears that were included in the fear of crime measure. From previous research, it is evident that different types of crime can evoke different degrees of fear [24]. As an important contribution to previous studies, our study takes into consideration fear of types of offences relevant for individuals of advanced age. Overall, this result does not provide evidence for the hypothesized role of adaptive emotion regulation as a protective factor for maintaining higher levels of well-being despite experiencing fear of crime. 

### Strengths and Limitations

The main strength of the present study includes the focus on advanced age using a sample with a wide age range (oldest participant being 106 years of age) and using a clear definition of fear of crime. We utilized specific, age-relevant fears and validated scales to measure the main study variables (i.e., cognitive emotional regulation and depressive feelings). As always, our findings must be interpreted in the context of several limitations. The study relies on cross-sectional survey data; therefore, conclusions about the direction of the effect in the associations between fear of crime and emotion regulation and fear of crime and the subjective well-being outcomes are limited to statistical prediction. Following previous research [3,34,35], in this study, we conceptualized depressive feelings and lower life satisfaction as potential negative psychological effects of fear of crime and cognitive emotion regulation strategies as a personal strength or vulnerability factor. However, both fear of crime leading to decreases in health outcomes and reduced health exacerbating fear of crime should be considered [2,31,63]. For instance, it was demonstrated that depression statistically predicts fear of crime in advanced age [24]. Moreover, a longitudinal study on fear of crime and mental health suggested stronger evidence for psychological distress having an effect on increased fear of crime over time than for the reversed direction [63]. Similarly, being prone to maladaptive cognitive emotion regulation could be a plausible consequence of frequent and intense fear of crime experiences and therefore bi-directional associations between these factors could be tested further in longitudinal research. 

In addition, our sample was limited to the residents of senior apartments. Therefore, generalizations to the entire population of older adults should be made with caution because fear of crime for homeowners or for those residing in special-care settings could manifest differently. Because only the Swedish and the English language versions of the study questionnaire were available, only participants who could respond in Swedish or in English were included in the study. Moreover, lower reliability of certain cognitive emotion regulation subscales as well as a positive correlation of rumination and self-blame with acceptance warrants further replication of the study findings in different samples of older adults in order to corroborate the research results. 

## 5. Conclusions

To summarize, the study corroborated the previous research results in showing the associations between fear of crime and depressive feelings and life satisfaction in advanced age. The study contributed to the growing line of research on psychological vulnerability factors in the experience of fear of crime [45,47,48]. Specifically, our results highlight the importance of rumination, blaming others, and catastrophizing as cognitive emotion regulation strategies associated with affective fear of crime. Furthermore, we observed that rumination and self-blame strengthened the negative association between fear of crime and life satisfaction among older adults. Our findings articulate the detrimental role of these maladaptive cognitive emotion regulation strategies as individual psychological vulnerability factors in the experience of fear of crime. Moreover, this conclusion has important practical implications considering that emotion regulation can improve throughout life [64]. Interventions aimed at reducing the use of these maladaptive cognitive emotion regulation strategies may reduce the negative effect of fear of crime on well-being outcomes in older adults. On the contrary, acceptance, positive refocus, and putting into perspective were neither associated with affective fear of crime nor buffered the negative effect of fear of crime on well-being outcomes. Hence, these adaptive cognitive emotion regulation strategies cannot, based on the current study, be considered to be protective factors in relation to fear of crime and its consequences for well-being among older adults. 

## Figures and Tables

**Table 1:** Descriptive statistics for the study variables.

Variable	Min	Max	*M*	*SD*	*N*
1. Affective fear of crime	1.00	4.00	1.62	0.60	554
2. Depressive feelings	0.00	3.00	0.56	0.45	593
3. Life satisfaction	1.00	7.00	4.66	1.28	561
4. Self-blame	1.00	5.00	2.18	0.92	578
5. Rumination	1.00	5.00	2.53	0.97	570
6. Blaming others	1.00	5.00	1.83	0.85	577
7. Catastrophizing	1.00	5.00	1.84	0.84	580
8. Acceptance	1.00	5.00	3.28	1.09	575
9. Positive refocus	1.00	5.00	2.55	0.98	580
10. Putting into perspective	1.00	5.00	2.89	0.99	576

**Table 2:** Zero-order correlations (below diagonal) and partial correlations (above diagonal) controlling for age and gender, among the study variables.

Variable	1	2	3	4	5	6	7	8	9	10
1. Affective fear of crime	-	0.283 **	−0.284 **	0.036	0.099 *	0.126 **	0.265 **	−0.039	−0.006	−0.022
2. Depressive feelings	0.246 **	-	−0.545 **	0.026	0.057	0.084	0.228 **	−0.126 **	−0.180 **	−0.139 **
3. Life satisfaction	−0.277 **	−0.537 **	-	−0.001	0.026	−0.017	−0.166 **	0.204 **	0.236 **	0.137 **
4. Self-blame	0.027	0.033	−0.003	-	0.401 **	−0.040	0.205 **	0.337 **	0.215 **	0.301 **
5. Rumination	0.101 *	0.055	0.028	0.396 **	-	0.141 **	0.329 **	0.441 **	0.250 **	0.302 **
6. Blaming others	0.099 *	0.102 *	−0.023	−0.027	0.129 **	-	0.346 **	−0.023	0.120 **	0.061
7. Catastrophizing	0.255 **	0.236 **	−0.160 **	0.198 **	0.333 **	0.322 **	-	0.005	0.065	0.032
8. Acceptance	−0.032	−0.125 **	0.206 **	0.330 **	0.444 **	−0.037	0.017	-	0.285 **	0.372 **
9. Positive refocus	−0.003	−0.175 **	0.237 **	0.212 **	0.253 **	0.109 *	0.072	0.288 **	-	0.447 **
10. Putting into perspective	−0.020	−0.133 **	0.139 **	0.296 **	0.305 **	0.050	0.044	0.376 **	0.449 **	-

** *p* < 0.01, * *p* < 0.05; listwise N = 477.

**Table 3:** Summary of regression analyses for the models predicting depressive feelings.

Variable	*B*	SE	*β*	95% CI		
				*LL*	*UL*	*p*
Self-blame						
Age	0.011	0.003	0.169	0.005	0.016	<0.001
Gender ^1^	0.139	0.038	0.154	0.063	0.214	<0.001
Affective fear of crime	0.195	0.031	0.266	0.134	0.257	<0.001
Self-blame	0.009	0.021	0.018	−0.032	0.049	0.669
Affective fear of crime × self-blame	−0.013	0.034	−0.016	−0.080	0.053	0.700
R^2^	0.107					
Adjusted R^2^	0.098					
Rumination						
Age	0.010	0.003	0.156	0.005	0.015	<0.001
Gender ^1^	0.143	0.039	0.159	0.067	0.219	<0.001
Affective fear of crime	0.201	0.032	0.275	0.138	0.264	<0.001
Rumination	0.017	0.019	0.037	−0.022	0.055	0.392
Affective fear of crime × rumination	−0.040	0.030	−0.058	−0.098	0.018	0.175
R^2^	0.108					
Adjusted R^2^	0.099					
Blaming others						
Age	0.011	0.003	0.170	0.005	0.016	<0.001
Gender ^1^	0.128	0.039	0.143	0.052	0.205	0.001
Affective fear of crime	0.197	0.032	0.268	0.134	0.259	<0.001
Blaming others	0.043	0.022	0.083	−0.001	0.086	0.055
Affective fear of crime × blaming others	−0.045	0.033	−0.058	−0.110	0.020	0.172
R^2^	0.117					
Adjusted R^2^	0.108					
Catastrophizing						
Age	0.009	0.003	0.142	0.004	0.014	0.001
Gender ^1^	0.143	0.038	0.160	0.070	0.217	<0.001
Affective fear of crime	0.166	0.032	0.227	0.102	0.230	<0.001
Catastrophizing	0.099	0.024	0.186	0.053	0.145	<0.001
Affective fear of crime × catastrophizing	−0.052	0.034	−0.067	−0.118	0.015	0.126
R^2^	0.135					
Adjusted R^2^	0.126					
Acceptance						
Age	0.010	0.003	0.155	0.005	0.015	<0.001
Gender ^1^	0.138	0.038	0.153	0.062	0.213	<0.001
Affective fear of crime	0.197	0.031	0.268	0.136	0.258	<0.001
Acceptance	−0.041	0.017	−0.099	−0.075	−0.007	0.019
Affective fear of crime × acceptance	−0.046	0.027	−0.072	−0.100	0.007	0.087
R^2^	0.117					
Adjusted R^2^	0.108					
Positive refocus						
Age	0.011	0.003	0.169	0.005	0.016	<0.001
Gender ^1^	0.135	0.038	0.150	0.061	0.209	<0.001
Affective fear of crime	0.191	0.031	0.261	0.130	0.251	<0.001
Positive refocus	−0.081	0.019	−0.182	−0.119	−0.044	<0.001
Affective fear of crime × positive refocus	−0.031	0.034	−0.039	−0.098	0.036	0.361
R^2^	0.134					
Adjusted R^2^	0.126					
Putting into perspective						
Age	0.011	0.003	0.182	0.006	0.017	<0.001
Gender ^1^	0.137	0.038	0.153	0.062	0.211	<0.001
Affective fear of crime	0.198	0.031	0.272	0.137	0.259	<0.001
Putting into perspective	−0.052	0.019	−0.118	−0.089	−0.016	0.005
Affective fear of crime × putting into perspective	−0.010	0.031	−0.014	−0.071	0.051	0.747
R^2^	0.126					
Adjusted R^2^	0.117					

*Note*. ^1^ 1 = female, 2 = male. CI = confidence interval; *LL* = lower limit; *UL* = upper limit.

**Table 4:** Summary of regression analyses for the models predicting life satisfaction.

Variable	*B*	SE	*β*	95% CI		
				*LL*	*UL*	*p*
Self-blame						
Age	−0.002	0.008	−0.013	−0.018	0.013	0.770
Gender ^1^	−0.203	0.115	−0.078	−0.429	0.022	0.077
Affective fear of crime	−0.609	0.096	−0.279	−0.798	−0.420	<0.001
Self-blame	0.005	0.061	0.004	−0.115	0.126	0.933
Affective fear of crime × self-blame	−0.216	0.102	−0.092	−0.415	−0.016	0.034
R^2^	0.086					
Adjusted R^2^	0.076					
Rumination						
Age	−0.004	0.008	−0.020	−0.019	0.012	0.638
Gender ^1^	−0.187	0.114	−0.072	−0.411	0.036	0.101
Affective fear of crime	−0.631	0.095	−0.290	−0.818	−0.443	<0.001
Rumination	0.046	0.058	0.034	−0.068	0.161	0.429
Affective fear of crime × rumination	−0.190	0.091	−0.090	−0.370	−0.011	0.038
R^2^	0.091					
Adjusted R^2^	0.081					
Blaming others						
Age	−0.003	0.008	−0.017	−0.019	0.013	0.691
Gender ^1^	−0.199	0.117	−0.076	−0.429	0.032	0.091
Affective fear of crime	−0.626	0.097	−0.287	−0.817	−0.434	<0.001
Blaming others	−0.004	0.068	−0.002	−0.137	0.130	0.956
Affective fear of crime × blaming others	0.045	0.100	0.020	−0.150	0.241	0.649
R^2^	0.081					
Adjusted R^2^	0.071					
Catastrophizing						
Age	0.001	0.008	0.004	−0.015	0.017	0.926
Gender ^1^	−0.203	0.113	−0.078	−0.426	0.020	0.074
Affective fear of crime	−0.563	0.099	−0.258	−0.758	−0.368	<0.001
Catastrophizing	−0.216	0.071	−0.140	−0.355	−0.077	0.002
Affective fear of crime × catastrophizing	0.116	0.109	0.048	−0.098	0.330	0.287
R^2^	0.098					
Adjusted R^2^	0.089					
Acceptance						
Age	−0.005	0.008	−0.026	−0.020	0.011	0.536
Gender ^1^	−0.165	0.112	−0.063	−0.386	0.055	0.142
Affective fear of crime	−0.607	0.093	−0.279	−0.790	−0.423	<0.001
Acceptance	0.211	0.051	0.177	0.111	0.310	<0.001
Affective fear of crime × acceptance	−0.042	0.082	−0.022	−0.204	0.119	0.607
R^2^	0.113					
Adjusted R^2^	0.104					
Positive refocus						
Age	−0.003	0.008	−0.018	−0.019	0.012	0.665
Gender ^1^	−0.180	0.111	−0.069	−0.399	0.039	0.107
Affective fear of crime	−0.620	0.093	−0.285	−0.803	−0.438	<0.001
Positive refocus	0.302	0.056	0.231	0.192	0.412	<0.001
Affective fear of crime × positive refocus	−0.080	0.101	−0.034	−0.279	0.119	0.432
R^2^	0.138					
Adjusted R^2^	0.129					
Putting into perspective						
Age	−0.005	0.008	−0.028	−0.021	0.010	0.517
Gender ^1^	−0.165	0.113	−0.063	−0.387	0.057	0.145
Affective fear of crime	−0.614	0.094	−0.284	−0.799	−0.429	<0.001
Putting into perspective	0.163	0.056	0.126	0.053	0.273	0.004
Affective fear of crime × putting into perspective	−0.044	0.093	−0.021	−0.228	0.139	0.635
R^2^	0.097					
Adjusted R^2^	0.088					

*Note*. ^1^ 1 = female, 2 = male. CI = confidence interval; *LL* = lower limit; *UL* = upper limit.

## Data Availability

Data are available on request due to ethical restrictions. The data are not publicly available due to respondent confidentiality.
